# Determinants of bacteriological outcomes in exacerbations of chronic obstructive pulmonary disease

**DOI:** 10.1007/s15010-015-0833-3

**Published:** 2015-09-14

**Authors:** S. Sethi, A. Anzueto, M. Miravitlles, P. Arvis, J. Alder, D. Haverstock, M. Trajanovic, R. Wilson

**Affiliations:** Division of Pulmonary, Critical Care and Sleep Medicine, University at Buffalo, State University of New York, Buffalo, NY USA; VA Medical Research, 151, 3495 Bailey Avenue, Buffalo, NY 14215 USA; South Texas Veterans Health Care System, University of Texas Health Science Center at San Antonio, San Antonio, TX USA; Pneumology Department, Hospital Universitari Vall d’Hebron, CIBER de Enfermedades Respiratorias (CIBERES), Barcelona, Spain; Bayer HealthCare, Loos, France; Bayer HealthCare Pharmaceuticals, Whippany, NJ USA; Bayer Inc., Toronto, ON Canada; Host Defence Unit, Royal Brompton Hospital, London, UK

**Keywords:** Bacteriological outcomes, Beta-lactams, Exacerbation of chronic obstructive pulmonary disease, Fluoroquinolones, Risk factors, Systemic corticosteroids

## Abstract

**Purpose:**

Changes in sputum microbiology following antibiotic treatment of acute exacerbations of chronic obstructive pulmonary disease (AECOPD), including patterns of bacteriological relapse and superinfection are not well understood. Sputum microbiology at exacerbation is not routinely performed, but pathogen presence and species are determinants of outcomes. Therefore, we determined whether baseline clinical factors could predict the presence of bacterial pathogens at exacerbation. Bacterial eradication at end of treatment (EOT) is associated with clinical resolution of exacerbation. We determined the clinical, microbiological and therapeutic factors that were associated with bacteriological eradication in AECOPD at EOT and in the following 8 weeks.

**Methods:**

Sputum bacteriological outcomes (i.e., eradication, persistence, superinfection, reinfection) from AECOPD patients (*N* = 1352) who were randomized to receive moxifloxacin or amoxicillin/clavulanate in the MAESTRAL study were compared. Independent predictors of bacterial presence in sputum at exacerbation and determinants for bacteriological eradication were analyzed by logistic regression and receiver operating characteristic (ROC) analyses.

**Results:**

Significantly greater bacteriological eradication with moxifloxacin was mainly driven by superior *Haemophilus influenzae* eradication (*P* = 0.002, EOT). Baseline clinical factors were a weak predictor of the presence of pathogens in sputum (AUC_ROC_ = 0.593). On multivariate analysis, poorer bacterial eradication was associated with antibiotic resistance (*P* = 0.0001), systemic steroid use (*P* = 0.0024) and presence of *P. aeruginosa* (*P* = 0.0282).

**Conclusions:**

Since clinical prediction of bacterial presence in sputum at AECOPD is poor, sputum microbiological analysis should be considered for guiding antibiotic therapy in moderate-to-severe AECOPD, particularly in those who received concomitant systemic corticosteroids or are at risk for infection with antibiotic-resistant bacteria.

**Electronic supplementary material:**

The online version of this article (doi:10.1007/s15010-015-0833-3) contains supplementary material, which is available to authorized users.

## Introduction

Exacerbations of chronic obstructive pulmonary disease (COPD) lead to a progressive decline in lung function, with even a single episode of exacerbation having a prolonged effect on health status [1]. Ultimately, 5-year survival is significantly reduced in patients who experience frequent exacerbations of COPD, particularly if they require hospitalization [2]. Exacerbations can be of bacterial, viral or non-infectious etiology, and a significant proportion can be multi-factorial [3, 4].

The controversy regarding the use of antibiotics in the treatment of acute exacerbations of COPD (AECOPD) has now been largely resolved, with increasing evidence of bacterial causation and of clinical benefits with antibiotic treatment [5, 6]. Moxifloxacin (MXF) and amoxicillin/clavulanic acid (AMC) are currently recommended antibiotics for the treatment of AECOPD in patients at risk for poor outcome [5, 7, 8]. These two antibiotics demonstrated similar clinical efficacy in the recently completed MAESTRAL trial in COPD outpatients with Anthonisen type I exacerbations [9].

Several novel observations were made in the MAESTRAL study. Approximately 50 % of patients had a pathogen isolated at baseline, and in this subgroup moxifloxacin was superior to amoxicillin/clavulanate in reducing clinical failures and relapses over the 8-week post-treatment period [9]. This may be the result of significantly higher bacteriological eradication rate at the end of antibiotic treatment with moxifloxacin compared with amoxicillin/clavulanic acid [9].

Although the efficacy of systemic corticosteroids is well established in hospitalized AECOPD patients, their role in outpatients remains unclear. Corticosteroids may influence clearance of bacteria by the immune system and result in incomplete eradication of pathogens leading to relapses. In MAESTRAL, higher clinical failure rate was observed in patients with co-administration of systemic corticosteroids compared with those who did not receive systemic steroids [9].

Additional analyses of the MAESTRAL database presented here were done to explore these interesting observations further. We analyzed in detail changes in microbiology after an AECOPD during the 8-week study period, including patterns of bacteriological relapse and superinfection. We aimed to identify the baseline clinical factors (in addition to sputum purulence) which could predict the presence of bacteria in sputum at exacerbation and thus guide antibiotic treatment choices in AECOPD. We investigated which baseline and therapy factors were associated with bacteriological outcomes at EOT and 8 weeks post-therapy. We analyzed further the relationship between sputum microbiology and clinical outcome, especially for *Pseudomonas aeruginosa*.

We also investigated whether there was an interaction between systemic corticosteroid use and sputum microbiology that may account for the higher clinical failure rate in corticosteroid-treated patients.

## Patients and methods

### Study design

As previously reported, MAESTRAL [NCT00656747] was a prospective, multinational, multicenter, randomized, double-blind, double-dummy, controlled study comparing the efficacy of 5 days of MXF 400 mg orally (PO) once-daily with 7 days of AMC 875/125 mg PO twice daily in outpatients experiencing an AECOPD [9]. Prior to randomization, patients were stratified according to the co-administration of oral corticosteroids (CS) for the current AECOPD, prescribed at the treating physician’s discretion. Full details of the study design, protocol, primary and secondary outcomes and definitions of clinical response have been reported previously [9, 10]. The primary endpoint was clinical failure rate at 8 weeks post-therapy in the per-protocol (PP) population [9]. Clinical failure was defined as the requirement for additional or alternate treatment with systemic antibiotics and/or systemic corticosteroids (including increased dose or duration of treatment), and/or hospitalization within 8 weeks post-therapy for an exacerbation of respiratory symptoms [9].

### Patient populations

Full details of the populations assessed in the MAESTRAL study have been published previously [9]. Briefly, patients, aged ≥60 years, had moderate-to-severe COPD, forced expiratory volume in 1 s (FEV_1_) ≤60 %, ≥2 exacerbations in the past 12 months and an Anthonisen type I exacerbation (i.e., increased dyspnea, increased volume and purulence of sputum). The populations assessed in this analysis were the PP with pathogens [all patients valid for the PP population with at least one pre-therapy potentially pathogenic bacteria (PPB) in sputum), ITT with pathogens (all patients valid for the ITT population with at least one pre-therapy PPB in sputum), and the complete ITT population.

### Microbiology

Spontaneous sputum samples, obtained from all patients at enrolment and each subsequent clinic visit (if obtainable), were assessed in a local laboratory by Gram stain and culture. The investigator had to confirm macroscopically the purulence of the sputum sample and also graded it as either yellow or green or rust (according to the provided color chart). Microscopic quality assessments of all samples were carried out, and squamous epithelial and polymorphonuclear cell counts were assessed semi-quantitatively. Pure sub-cultures of PPB which were predefined in the study protocol (*Haemophilus* spp., *Streptococcus pneumoniae, Moraxella catarrhalis, P. aeruginosa, Klebsiella* spp., *Enterobacteriaceae* spp., and *Staphylococcus aureus*) isolated on culture from these sputum samples were then frozen and forwarded to a central microbiology laboratory. There, the identity of bacteria was confirmed followed by determination of minimum inhibitory concentrations (MICs) for MXF, AMC, and a series of other antibiotics by the reference Clinical Laboratory Standards Institute broth dilution method [11].

MICs for penicillin were determined for *S. pneumoniae* and *S. aureus* isolates were tested for oxacillin susceptibility to determine methicillin-resistant *S. aureus* (MRSA) status. Beta-lactamase production for *H. influenzae* was classified by the ampicillin MIC, with a value of ≥2 mg/L considered positive.

Bacteriological response rates were assessed during therapy, at EOT and at 4 and 8 weeks post-therapy. Bacteriological responses to therapy were defined as the following: (1) bacteriological eradication without superinfection or reinfection—initial causative pathogen(s) absent, no new pathogen isolated after start of study; (2) presumed eradication—absence of appropriate culture material for evaluation because the subject has clinically improved on therapy; (3) persistence—initial causative pathogen(s) still present; (4) presumed persistence—absence of appropriate culture material for evaluation in a subject who has not clinically improved on therapy; (5) bacteriological eradication with superinfection—initial causative pathogen(s) absent, a new pathogen isolated during treatment or at EOT; (6) bacteriological eradication with reinfection—initial causative pathogen(s) absent, a new pathogen isolated after EOT; (7) eradication with recurrence—original causative organism absent at EOT, but reappearance of the same organism at or before 8 weeks post-therapy; (8) continued eradication—the causative organism(s) is absent at this time point; (9) continued presumed eradication—the absence of appropriate culture material for evaluation because the patient has clinically improved; (10) indeterminate—bacterial response to the study drug was not evaluable. Bacteriological success was defined as the sum of confirmed and presumed eradication of bacteria. Bacteriological failure was defined as the sum of persistence, presumed persistence, superinfection, reinfection and indeterminate outcomes.

### Statistical analyses

Bacteriological outcomes were summarized by bacterial species and treatment group using descriptive statistics. Bacteriological outcomes by subject in the MXF and AMC arms were compared to test non-inferiority (at 6 % level with Mantel–Haenszel test).

Clinical and bacteriological outcomes in patients with *Pseudomonas aeruginosa* isolated from sputum at baseline were compared with those without this pathogen. The interaction of PPB presence in sputum and systemic steroid use at the different time points with regard to clinical failure rate was analyzed by one-way ANOVA.

Pre-therapy factors (see Supplementary Table 1; i.e., demographic, medical history, anamnesis related to exacerbation, medications, sputum characteristics, organisms related to exacerbation, AECB-SS questionnaire details) were analyzed using univariate and multivariate logistic regression analyses to evaluate their association with the presence of PPB in sputum at enrolment and with bacteriological endpoints. A receiver operating characteristic (ROC) analysis was also performed to determine whether the combination of the independent risk factors would provide a reliable model to predict the presence of pathogens in the airways during an acute exacerbation. The independent predictors for presence of bacteria were also evaluated in terms of their frequency. For categorical variables *P* value calculation was based on the presence or absence (or predefined categories) of the variable. For continuous variables *P* value calculation was based on individual values of the independent variable.

Pre-therapy factors were combined with on therapy factors to determine their relationship with bacteriological endpoints, by univariate and multivariate logistic regression analyses.

## Results

### Bacteriological results

#### Bacteriological demographics and etiology

Of the identified potential pathogens at baseline in *N* = 662 patients, 23.4 % were Gram-positive, 35.3 % were *Enterobacteriaceae* and 59.8 % were other Gram-negative (Table 1). Patients could have more than one isolated species at baseline (monomicrobial infection: 82.6 %; 547/662 patients; polymicrobial infection: 17.4 %; 115/662 patients). The pattern of frequency and types of the most frequent isolates were similar in both treatment groups.

**Table 1:** Distribution of all baseline potential pathogens by rank order grouping of species and main species (ITT with pathogens population, *N* = 662)

Species	Total *n*/*N* (%)	Moxifloxacin *n*/*N* (%)	Amoxicillin/clavulanic acid *n*/*N* (%)
Total_patients_	*N* = 662	*N* = 327	*N* = 335
Total_bacteria_	*N* = 785	*N* = 385	*N* = 400
Gram-positive			
Total (% of patients)^a^	155 (23.4)	80 (24.5)	75 (22.4)
***Streptococcus pneumoniae***	**87** (**13.1**)	**49** (**15.0**)	**38** (**11.3**)
*Staphylococcus aureus*	43 (6.5)	23 (7.0)	20 (6.0)
*Streptococcus* spp.	8 (1.2)	3 (0.9)	5 (1.5)
*Streptococcus viridans* group	5 (0.8)	1 (0.3)	4 (1.2)
*Streptococcus agalactiae*	4 (0.6)	2 (0.6)	2 (0.6)
*Streptococcus pyogenes*	3 (0.5)	0 (0)	3 (0.9)
*Streptococcus mitis*	1 (0.2)	1 (0.3)	0 (0)
*Enterococcus faecalis*	1 (0.2)	1 (0.3)	0 (0)
*Enterococcus faecium*	1 (0.2)	0 (0)	1 (0.3)
*Staphylococcus epidermidis*	1 (0.2)	0 (0)	1 (0.3)
*Staphylococcus haemolyticus*	1 (0.2)	0 (0)	1 (0.3)
Gram-negative non-*Enterobacteriaceae*			
Total (% of patients)^a^	396 (59.8)	191 (58.4)	205 (61.2)
***Haemophilus influenzae***	**140** (**21.1**)	**65** (**19.9**)	**75** (**22.4**)
***Pseudomonas aeruginosa***	**111** (**16.8**)	**57** (**17.4**)	**54** (**16.1**)
***Moraxella catarrhalis***	**79** (**11.9**)	**36** (**11.0**)	**43** (**12.8**)
*Acinetobacter baumannii*	18 (2.7)	12 (3.7)	6 (1.8)
*Haemophilus parainfluenzae*	18 (2.7)	5 (1.5)	13 (3.9)
*Stenotrophomonas maltophilia*	8 (1.2)	2 (0.6)	6 (1.8)
*Acinetobacter lwoffii*	4 (0.6)	3 (0.9)	1 (0.3)
*Morganella morganii*	3 (0.5)	3 (0.9)	0 (0.0)
*Haemophilus* spp.	3 (0.5)	2 (0.6)	1 (0.3)
*Burkholderia cepacia*	2 (0.3)	1 (0.3)	1 (0.3)
*Moraxella* spp.	2 (0.3)	0 (0)	2 (0.6)
*Moraxella osloensis*	2 (0.3)	2 (0.6)	0 (0.0)
*Pseudomonas fluorescens*	1 (0.2)	0 (0.0)	1 (0.3)
*Pseudomonas putida*	1 (0.2)	0 (0.0)	1 (0.3)
*Alcaligenes xylosoxidans*	1 (0.2)	0 (0.0)	1 (0.3)
*Haemophilus haemolyticus*	1 (0.2)	1 (0.3)	0 (0.0)
*Haemophilus parahaemolyticus*	1 (0.2)	1 (0.3)	0 (0.0)
*Bordetella bronchiseptica*	1 (0.2)	1 (0.3)	0 (0.0)
*Enterobacteriaceae*			
Total (% of patients)^a^	234 (35.3)	114 (34.9)	120 (35.8)
***Klebsiella pneumoniae***	**84** (**12.7**)	**36** (**11.0**)	**48** (**14.3**)
*Escherichia coli*	37 (5.6)	21 (6.4)	16 (4.8)
*Serratia marcescens*	28 (4.2)	14 (4.3)	14 (4.2)
*Enterobacter cloacae*	19 (2.9)	11 (3.4)	8 (2.4)
*Enterobacter aerogenes*	16 (2.4)	8 (2.4)	8 (2.4)
*Klebsiella oxytoca*	15 (2.3)	11 (3.4)	4 (1.2)
*Proteus mirabilis*	13 (2.0)	4 (1.2)	9 (2.7)
*Citrobacter koseri*	13 (2.0)	6 (1.8)	7 (2.1)
*Proteus vulgaris*	2 (0.3)	0 (0.0)	2 (0.6)
*Citrobacter freundii*	2 (0.3)	1 (0.3)	1 (0.3)
*Citrobacter amalonaticus*	2 (0.3)	1 (0.3)	1 (0.3)
*Klebsiella ozaenae*	1 (0.2)	0 (0.0)	1 (0.3)
*Enterobacter hormaechei*	1 (0.2)	1 (0.3)	0 (0.0)
*Serratia liquefaciens*	1 (0.2)	0 (0.0)	1 (0.3)

*P* values were >0.05 for all potential pathogens; species >10 % of total are bolded

*n* number of patients with potential pathogens isolated at baseline, *N* total number of patients, *ITT* intent-to-treat, *spp* species

^a^Total number of patients with potential pathogen group at baseline

#### Bacterial persistence

The most frequent persisting pathogens over the course of the study period in either MXF or AMC group were *P. aeruginosa*, *E. coli*, *K. pneumoniae*, *S. marcescens*, *M. catarrhalis* and *A. baumannii*. Among these persisting pathogens, the frequency of *H. influenzae* isolation was lower in MFX group compared with AMC group during the entire study (*P* < 0.05 at EOT; Supplementary Table 2).

#### Superinfections and reinfections

A total of 148 patients had superinfection up to EOT, 65 (9.6 % of 677 ITT patients) in the MXF group and 83 (12.3 % of 675 ITT patients) in the AMC group (*P* = 0.11) (see Supplementary Table 3 for detailed listing of pathogens). The most common superinfecting organisms (≥10 % of total number of superinfecting bacteria) in both groups were *P. aeruginosa, K. pneumoniae, H. influenzae, A. baumannii*, *M. catarrhalis, S. aureus, E. coli, E. cloacae, K. oxytoca, S. pneumoniae* and *S. marcescens*. Among these pathogens, significant differences between the two arms (MXF vs AMC) were seen for *H. influenzae* (0.9 vs 2.7 %, *P* = 0.013), *K. oxytoca* (0.2 vs 2.1 %, *P* < 0.001) and *E. cloacae* (0.6 vs 2.1 %, *P* = 0.017), respectively.

A total of 119 reinfections were observed up to 8 weeks post-therapy, and the rate was very similar in the two treatment groups [*n* = 58 (8.6 %) in MXF group and *n* = 61 (9.0 %) in AMC group].The most common (≥10 % of total number of reinfecting bacteria) species were *P. aeruginosa*, *K. pneumoniae, S. pneumoniae, M. catarrhalis, H. influenzae, K. oxytoca, E. coli, E. cloacae* and *S. marcescens* (Supplementary Table 4). A significant difference between the two arms (MXF vs AMC) was seen for *H. influenzae* (1.0 vs 2.8 %, *P* = 0.017), while numerical (not significant) differences were seen for *M. catarrhalis* (2.4 vs 1.2 %), *S. aureus* (1.0 vs 0.3 %), *K. oxytoca* (0.7 vs 1.6 %), *E. coli* (1.2 vs 0.6 %) and *E. cloacae* (0.4 vs 1.3 %).

#### In vitro susceptibility of persisting, reinfecting and superinfecting pathogens

MIC changes during therapy or up to 8 weeks post-therapy were rare in persisting or reinfecting organisms and were minor in both treatment groups. Susceptibility patterns of superinfecting organisms did not differ from those of pre-therapy pathogens (Supplementary Table 5).

#### Bacteriological outcomes by species

**Fig. 1:**
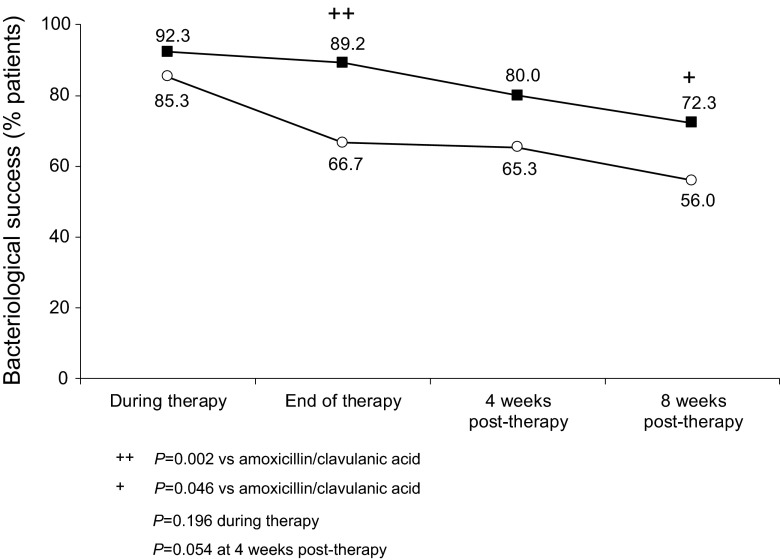
Bacteriological success^a^ of *H. influenzae* by timepoint (ITT with pathogens population, *N* = 662); *filled square* moxifloxacin; *unfilled circle* amoxicillin/clavulanic acid. ^a^Eradication and presumed eradication; *ITT* intent-to-treat

For individual pathogens, the most striking and consistent difference in bacteriological eradication between the 2 treatments was seen with *H. influenzae* (Fig. 1). The most pronounced difference in *H. influenzae* eradication rates between MXF and AMC was observed at EOT (89.2 vs 66.7 %, *P* = 0.002); however, over the 8-week post-therapy period the difference was maintained.

Changes in bacteriological success for the most prevalent 6 pathogens over the study period in the ITT with pathogens and PP with pathogens populations are provided in the Supplementary Material (Supplementary Table 6). There were no statistically significant differences observed between the 2 treatments for any other pathogens at any time point. A trend towards higher eradication rates for *P. aeruginosa* at 8 weeks post-therapy in the PP with pathogens population (*P* = 0.06) was seen with MXF compared with AMC. There was also a trend for eradication rates for *M. catarrhalis* to be higher in the AMC group than in the MXF group at 4 weeks (*P* > 0.1) and 8 weeks (*P* = 0.08) post-therapy in the ITT with pathogens population; this difference in eradication rate was seen in the PP with pathogens population only at 8 weeks post-therapy (Supplementary Table 6).

#### Predictive factors for bacterial isolation at enrolment

In the univariate analyses, several factors were found to be predictive of bacterial isolation from sputum at enrolment (*P* < 0.1, included in Supplementary Table 1). Multivariate logistic regression analysis revealed seven factors that were independently associated with the presence of PPB at enrolment (Table 2).

**Table 2:** Pre-therapy risk factors independently associated with isolation of potentially pathogenic bacteria at enrolment (ITT population, *N* = 1352)

Risk factors	Bacteria		*P* value^a^
	Present (%)	Absent (%)	
Age	≥65 years	<65 years	0.003
	51.6	42.0	
History of cardiopulmonary disease	Yes	No	0.016
	58.6	47.7	
FEV_1_ percent predicted	≥30 %	<30 %	0.0303
	50.6	44.0	
Sputum viscosity	Very thick	Not very thick	0.0147
	55.6	48.1	
Color of sputum (recorded as part of AECB-SS)	Green or brown	Other colors	0.0214
	54.4	47.0	
Wheeze present	Yes	No	0.0327
	50.4	43.0	
Anti-cholinergic use	Yes	No	0.0148
	57.9	47.7	

^a^Multivariate analysis

*AECB-SS* Acute Exacerbation of Chronic Bronchitis Symptom Scale, *FEV*_*1*_ forced expiratory volume in 1 s, *ITT* intent-to-treat

**Fig. 2:**
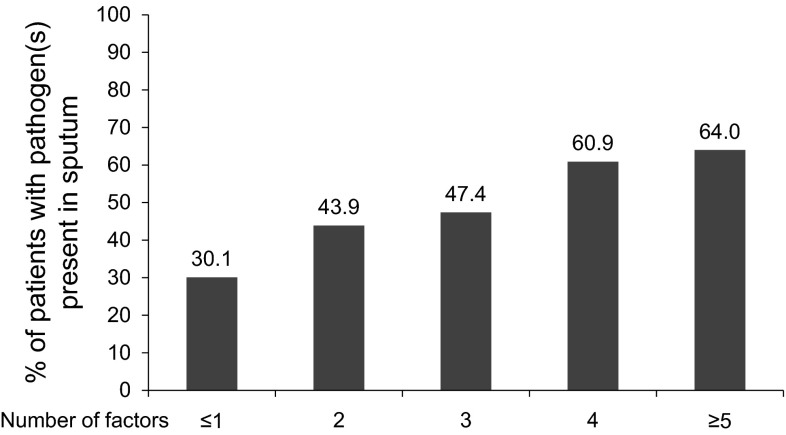
Risk of presence of bacteria in sputum based on number of risk factors (ITT with pathogens population, *N* = 662). *ITT* intent-to-treat

When the pre-therapy clinical risk factors were pooled, presence of at least four factors provided a probability of the presence of PPB at baseline of 60.9 % (173/284 patients) (Fig. 2). When five or more factors were present, the probability increased to 64.0 % (48/75 patients). There were only three patients with six factors and no patient with all seven factors present simultaneously. The ROC curve analysis suggested that the combination of these seven independent risk factors would poorly predict the presence of pathogens in the airways during an acute exacerbation (area under the curve (AUC) = 0.593].

#### Predictive factors at EOT and at 8 weeks post-therapy for confirmed bacteriological eradication from sputum and bacteriological success including presumed eradication

Using multivariate analyses, several pre- and on-therapy independent factors were found to be significantly associated with confirmed eradication of bacteria (vs lack of eradication) in the sputum at EOT and 8 weeks post-therapy.

**Fig. 3:**
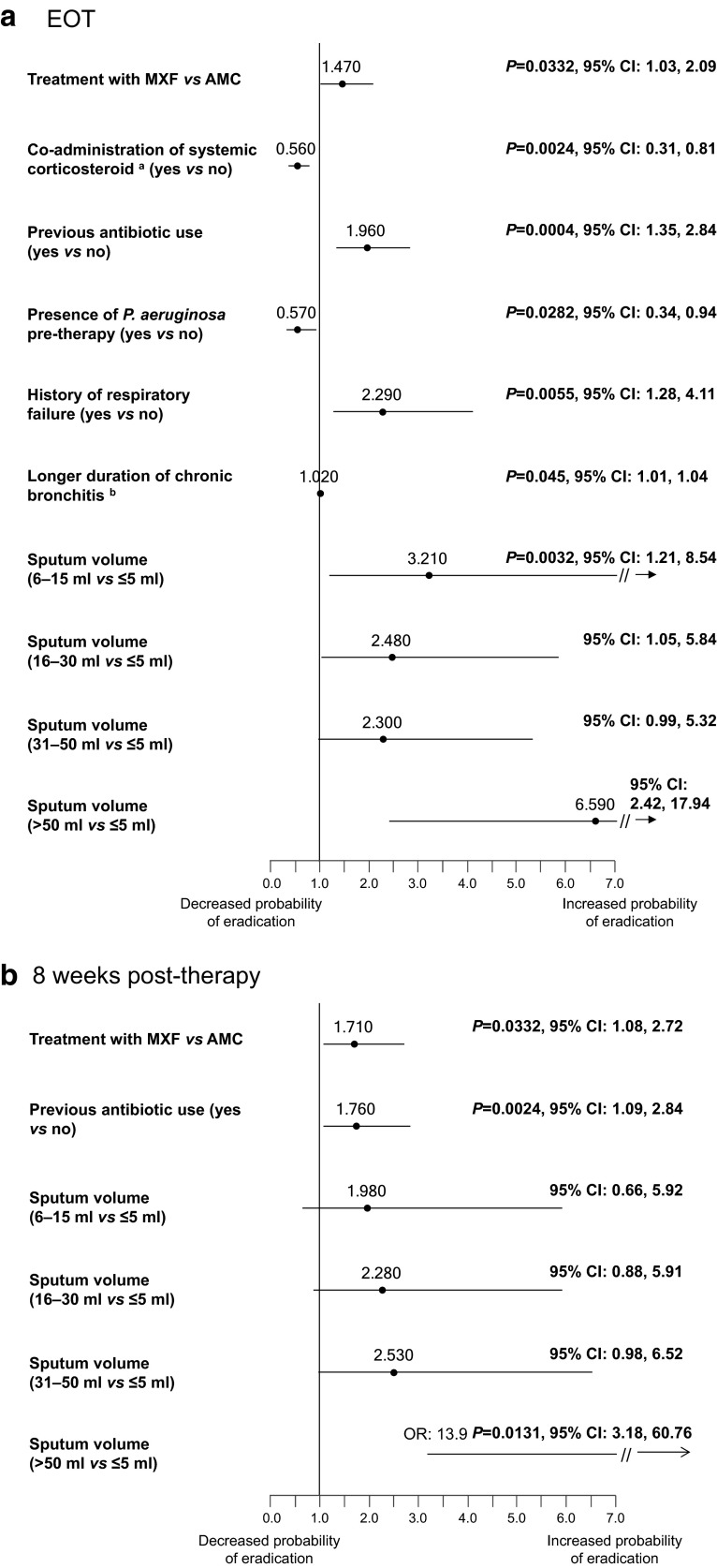
**a** Prognostic factors associated with confirmed bacterial eradication at end of therapy (ITT with pathogens population, *N* = 662). ^a^At current exacerbation, ^b^continuous value; *AMC* amoxicillin/clavulanic acid; *CI* confidence interval, *EOT* end of therapy, *ITT* intent-to-treat, *MXF* moxifloxacin. **b** Prognostic factors associated with confirmed bacteriological eradication at 8 weeks post-therapy (ITT with pathogens population, *N* = 662). *ITT* intent-to-treat, *CI* confidence interval, *OR* odds ratio

At EOT, patients who received MXF, who had been treated previously with a course of antibiotic in the last 3 months, had a history of respiratory failure or a duration of chronic bronchitis of ≥10 years were more likely to eradicate bacteria from their airways at EOT (Fig. 3a). However, patients who were also treated with systemic corticosteroids for the current exacerbation or were infected with *P. aeruginosa* were less likely to eradicate bacteria (Fig. 3a). Patients were more likely to remain pathogen free at 8 weeks post-therapy if they received MXF instead of AMC or had been treated previously with a course of antibiotic in the last 3 months (Fig. 3b). The analyses also revealed that higher volume of sputum was significantly associated with bacteriological success at EOT or up to 8 weeks post-therapy (Fig. 3a, b).

Additional analyses were conducted to assess factors related to bacteriological success (when, in addition to confirmed eradication, presumed eradication [see definition above] was also taken into account). In the multivariate analysis for EOT outcomes, patients with a body mass index (BMI) at normal range or above (e.g., ≥20 kg/m^2^) were more likely to achieve bacteriological success [odds ratio (OR): 1.05, *P* = 0.0044, 95 % confidence interval (CI) 1.02, 1.10]. However, bacteriological success was less likely with infection by pathogens resistant to study drugs (OR 0.45, *P* = 0.0001, 95 % CI 0.30, 0.68) or among patients treated with long-acting β-agonists (LABA) (OR 0.55, *P* = 0.0024, 95 % CI 0.37, 0.81). At 8 weeks post-therapy, patients with a body temperature ≥36 °C were more likely to achieve bacteriological success (OR 1.89, *P* < 0.0001, 95 % CI 1.37, 2.63). However, bacteriological success was less likely if patients were treated with LABA (OR 0.44, *P* < 0.0001, 95 % CI 0.29, 0.65) or systemic corticosteroids for the current exacerbation (OR 0.70, *P* = 0.0405, 95 % CI 0.50, 0.98).

#### Exacerbations with *P. aeruginosa* infection

Comparison of the baseline characteristics of patients with and without *P. aeruginosa* versus the overall ITT population showed no major differences (Table 3). Clinical failure at 8 weeks post-therapy in patients with *P. aeruginosa* was 21.1 % in the MXF and 29.6 % in the AMC treatment groups (*P* = 0.298), while clinical failure rates were similar in patients without *P. aeruginosa* infection (20.3 % in MXF and 20.9 % in AMC treatment groups, respectively, *P* = 0.790) (Supplementary Fig. 1).

**Table 3:** Demographics and baseline characteristics of patients with or without *P. aeruginosa* isolated at enrolment (ITT population, *N* = 1352)

Characteristics	With *P. aeruginosa* (*N* = 111)	Without *P. aeruginosa* (*N* = 1241)	All patients (*N* = 1352)
Male sex, *n* (%)	95 (85.6)	984 (79.3)	542 (79.8)
Age (years), mean ± SD	70.9 ± 7.3	69.4 ± 6.7	69.6 ± 6.7
Range	60–88	59–93	59–93
≥65 years, *n* (%)	85 (76.6)	893 (72.0)	978 (72.3)
BMI (kg/m^2^), mean ± SD	23.8 ± 4.9	25.0 ± 5.2	24.8 ± 5.3
Systemic corticosteroid use for current exacerbation, *n* (%)	45 (40.5)	430 (34.6)	475 (35.1)
Previous antimicrobial use, *n* (%)	36 (32.4)	426 (34.3)	462 (34.2)
Duration of chronic bronchitis (years), mean ± SD	9.6 ± 7.1	9.3 ± 7.5	9.4 ± 7.5
FEV_1_ (L), mean ± SD	0.96 ± 0.35	0.98 ± 0.37	0.98 ± 0.36^b^
FEV_1_, % predicted, mean ± SD	38.3 ± 11.25	38.6 ± 11.7	38.6 ± 11.6
FEV_1_ < 30 %, *n* (%)	29 (26.4)	310 (25.0)	339 (25.1)
FEV_1_ ≥ 30 %, *n* (%)	81 (73.6)^a^	927 (74.7)	1008 (74.6)
Cardiopulmonary disease, *n* (%)			
Yes	14 (12.6)	143 (11.5)	157 (11.6)
No	97 (87.4)	1098 (88.5)	1195 (88.4)
Exacerbations in previous year			
Mean ± SD	2.6 ± 1.5	2.5 ± 1.0	2.5 ± 1.1
Range	2–15	1–10	1–15

^a^ *N* = 110

^b^ *N* = 1347

*BMI* body mass index, *FEV*_*1*_ forced expiratory volume in 1 s, *ITT* intent-to-treat, *SD* standard deviation

#### Clinical outcome according to pathogen presence in sputum and systemic corticosteroid use

**Fig. 4:**
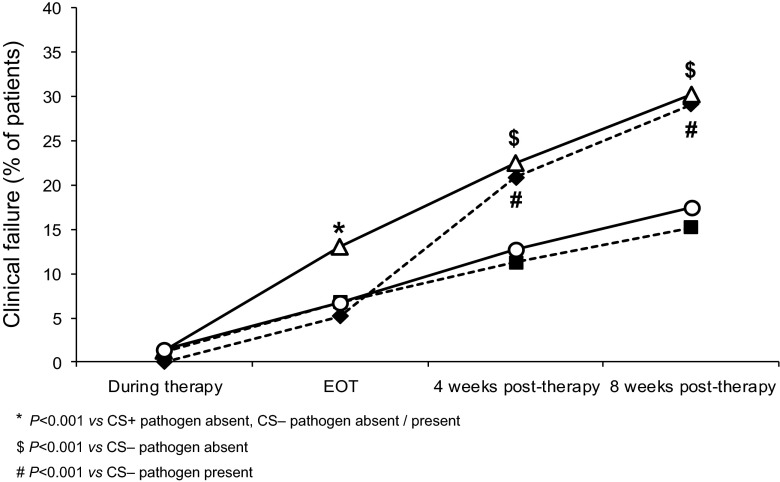
Clinical failure^a^ rates in patients over time with or without corticosteroid use and by causative pathogen presence or absence at enrolment in sputum (ITT population, *N* = 1352); *unfilled triangle* CS+ pathogen present (*n* = 245); *filled diamond* CS+ pathogen absent (*n* = 230); *unfilled circle* CS− pathogen present (*n* = 417); *filled square* CS− pathogen absent (*n* = 460). ^a^Failure and relapse; *CS* corticosteroid; *EOT* end of therapy; *ITT* intent-to-treat

We examined the effects of the interaction of systemic corticosteroid use (CS, + or −) and pathogen presence (P, + or −) in sputum on clinical outcomes at different time points. Four groups of patients were identified, CS+ P+, CS+ P−, CS− P+, CS− P−. At EOT, clinical failure rate in the CS+ P+ group was higher (*P* < 0.001) than in the other 3 groups. At 4 and 8 weeks post-therapy, both CS+ groups demonstrated worse clinical outcomes than CS− groups (4 weeks: *P* < 0.001; 8 weeks: *P* < 0.001, Fig. 4).

## Discussion

Clinical outcomes have been the focus of many recent AECOPD trials. The importance of bacteria in causing exacerbations and contributing to chronic inflammation in COPD is widely recognized; therefore, the dynamics of bacterial infection following an AECOPD and the impact of our treatment choices on these dynamics need to be better understood. The current bacteriological results of the MAESTRAL study, which was a randomized, double-blind, double-dummy, controlled trial enrolling 1352 AECOPD outpatients, has yielded important observations. Analyses of the bacteriological dataset, and correlating it with clinical variables, showed that (1) MXF treatment versus AMC treatment had significantly higher and sustained eradicaton of *Haemophilus influenzae*, which is the most prevalent bacterial pathogen in AECOPD; (2) the investigated pre-therapy clinical factors are poorly predictive of the presence of pathogens in sputum; (3) the presence of *Pseudomonas aeruginosa* in the airways is associated with lack of bacteriological eradication in patients who were treated with antibiotics according to current guidelines; (4) concomitant systemic corticosteroid treatment is associated with poorer bacteriological eradication and higher clinical failure rate; (5) LABA treatment was also associated with higher risk of bacteriological failure at the end of therapy and it was maintained up to 8 weeks post-therapy, and (6) the presence of organisms in sputum pre-therapy resistant to study drugs was more likely to lead to bacteriological failure.

The spectrum and frequency of pathogens isolated in the MAESTRAL study were consistent with what is expected in COPD patients with moderate-to-severe disease [3, 9, 12–16], and no unusual resistance patterns were identified [9]. However, a higher frequency of pathogen isolation in patients with Anthonisen type I exacerbations might have been expected. Of note, viral exacerbations were not investigated in the study. However, it is becoming clear that sputum cultures are relatively insensitive for detecting PPBs in these patients and the rate of detection may be substantially increased by the addition of molecular detection methods [15, 16]. Furthermore, with new molecular technologies that rapidly reveal the entire microbiome in the sputum or the airway samples of these patients [17], more accurate information on the infectious etiology is expected in the future [18].

Overall bacteriological success rates were higher for MXF vs AMC throughout the study with the difference between treatments being significant during therapy and at EOT [9]. Surprisingly, as shown in the current analyses this difference was mainly driven by significantly higher eradication of *H. influenzae* with MXF, a pathogen sensitive to both antibiotics in vitro. The better bacteriological efficacy of MXF in vivo could be due to its high intracellular activity, while AMC penetrates cells poorly [19, 20]. Intracellular localization of non-typeable *H. influenzae* has been described in COPD and chronic upper respiratory infections, and likely promotes persistence and recurrence of this pathogen [21].

Sputum cultures are not widely used in the management of AECOPD due to their low sensitivity and specificity and a long turnaround time (up to 48 h); therefore, purulent sputum has been advocated as a predictor of bacterial exacerbations and as a guide to the need for antibiotics [7, 8, 22, 23]. In the MAESTRAL study, in which all randomized patients had purulent sputum, MXF was superior to AMC when PPBs were present in the sputum [9]; however, both antibiotics were equally clinically effective in patients without PPBs. In our analysis, the additional prognostic factors besides sputum purulence that might have predicted a PPB in sputum were older age, history of cardiopulmonary disease, FEV_1_ percent predicted (>30 vs <30 %), sputum viscosity, green/brown sputum, wheeze at exacerbation and short-acting anti-cholinergic use. Although these were all statistically significant predictive factors (*P* < 0.1 in univariate analysis), the presence of any of these risk factors increased the pathogen isolation rate by a maximum of 10.9 %, and the highest bacterial isolation rate was still 58.6 %. Furthermore, the ROC analysis (AUC = 0.593) suggests that the combination of these risk factors remains inadequate in predicting the presence of pathogenic bacteria in the sputum. Further research on sputum or blood biomarkers clearly associated with bacterial exacerbations and/or more reliable tests of the presence of bacteria in the airways other than sputum culture are greatly needed to support diagnosis.

The MAESTRAL study is the first study to show that bacterial eradication at EOT translates into longer-term clinical success [9]. In this analysis, we aimed to identify independent predictors of bacteriological eradication and success. We have found that among pathogen-positive patients who were treated with moxifloxacin, who received a prior course of antibiotic in the last 3 months, who had respiratory failure in the past, who had higher sputum volume at baseline, who were not infected by *P. aeruginosa* or any other resistant pathogen at enrolment, who had BMI ≥20 kg/m^2^, and a body temperature ≥36 °C were more likely to achieve bacteriological eradication or success after antibiotic therapy. The surprising association between a prior course of antibiotic within the previous 3 months and confirmed eradication of bacteria may be the result of reduced overall bacterial load in the airways of patients that might translate into better clinical outcome from the subsequent exacerbation. The results of a previous placebo-controlled study in which AECOPD patients received six courses of moxifloxacin therapy in every 8 weeks had significantly lower odds of a subsequent exacerbation [24]. The positive association between history of respiratory failure and the probability of bacteriological eradication seems counterintuitive; however, these patients might have received more supportive and respiratory therapies.

The co-administration of systemic corticosteroids for the current exacerbation was associated with lack of confirmed bacteriological eradication from sputum at EOT and lack of bacteriological success at 8 weeks post-therapy. Similarly, patients with maintenance LABA therapy had a significantly lower likelihood to achieve bacteriological success at both EOT and 8 weeks post-therapy. It is possible that administration of oral corticosteroids for the current exacerbation and LABA therapy are markers of a more severe disease status in this analysis; and the more severe disease is linked with lower capability of antibiotics and the body’s immune system to eradicate bacterial pathogens [5]. Nevertheless, these observations in this patient population warrant further investigation given the widespread use of LABA in stable COPD and oral corticosteroids for exacerbations.

Our analysis of the interaction of presence of bacterial pathogens in sputum and systemic corticosteroid use revealed very interesting results. In patients not given oral corticosteroids, failure rates were linear and followed similar trends in pathogen positive and negative exacerbations. In corticosteroid-treated pathogen-positive patients, higher failure rates were observed at all time points. Conversely, in corticosteroid-treated patients with pathogen-negative exacerbations, failure rates remained low until EOT, then increased substantially to become similar to the corticosteroid-treated pathogen-positive patients and higher than patients not treated with corticosteroids. Though effective in relieving symptoms in exacerbations, corticosteroids have immunosuppressive effects that can interfere with clearance of infectious pathogens. Both innate and adaptive immune responses in the lung are required for pathogen clearance. Alveolar macrophages are key orchestrators for both innate and adaptive immune responses. Alveolar macrophages obtained from COPD patients have impaired phagocytic ability for, and suppressed cytokine response to, bacterial pathogens implicated in recurrent infections in COPD [25, 26]. In an in vivo mouse model of pneumococcal lung infection and in vitro in alveolar macrophages, Stolberg et al. demonstrated that corticosteroids increased the early bacterial burden and impaired the phagocytic killing of pneumococci by alveolar macrophages [27]. A 7-day prior glucocorticoid exposure substantially weakened the adaptive immune signature of human monocyte-derived macrophages following stimulation with lipopolysaccharide (LPS, endotoxin) in vitro [28]. Additional impairment by corticosteroids of the innate and adaptive responses of lung macrophages to pathogens, which are already compromised in COPD, is likely to be a major contributor to diminished pathogen clearance and could explain our findings. Interestingly, in a recent study by Bafadhel et al. patients without peripheral blood eosinophilia at exacerbation who had more neutrophils and higher concentration of sputum bacteria, had a higher failure rate when given systemic corticosteroids as compared with placebo [29]. These observations suggest that universal prescription of systemic corticosteroids for all severe AECOPD may not be optimal, especially when bacterial infection is a likely cause of the exacerbation.

Increased airway inflammation is present during an acute exacerbation and resolves with successful treatment [3, 30–33]. Evidence suggests that the link between bacterial eradication and clinical resolution is the reduction in the level of inflammation after bacterial eradication [32, 34, 35]. Persistence, superinfection or reinfection may also predispose patients for relapses leading to the vicious cycle between inflammation and infection [5]. For this reason, a high rate of bacterial eradication from the airways (sputum) of AECOPD patients should be a key goal of antibiotic therapy, and a focus on bacterial eradication rather than simply on clinical resolution needs to be considered. Our data showed significant association between higher baseline sputum volume and confirmed bacteriological eradication from the sputum at the end of antibiotic therapy. This significant association, however, was not present when presumed eradication was also analyzed in patients who were clinically cured at EOT and, therefore, could not provide sputum sample. Therefore, its significance in clinical practice remains elusive.

In summary, this paper presents a detailed analysis of microbiological outcomes in AECOPD, and reveals that *H. influenzae* may not be adequately eradicated by AMC. The study also showed that in patients with purulent sputum clinical factors weakly predicted the presence of pathogens in sputum. Additionally, bacterial eradication was found to be impaired by systemic steroid use, antibiotic resistance and presence of *P. aeruginosa*. These observations suggest that sputum analysis (or a more precise rapid bacterial diagnostic test in the future) may be indicated to help optimize antibiotic therapy in patients with Anthonisen type I exacerbations who do not require hospitalization, particularly if they are at risk of being infected with *P. aeruginosa* or they receive concomitant systemic corticosteroids.

## Electronic supplementary material

Supplementary material 1 (PDF 130 kb)

Supplementary material 2 (PDF 469 kb)
