# Supplementary material for: Determinants of bacteriological outcomes in exacerbations of chronic obstructive pulmonary disease
Source: Infection. 2015 Sep 14;44:65–76. doi: 10.1007/s15010-015-0833-3 (PMC4735236; doi:10.1007/s15010-015-0833-3)
Supplement: Supplementary file 1 — Supplementary material 1 (PDF 130 kb) [file 15010_2015_833_MOESM1_ESM.pdf]

Determinants of bacteriological outcomes in exacerbations of chronic obstructive pulmonary disease; *Infection*; **Sethi S** et al. Division of Pulmonary, Critical Care and Sleep Medicine, University at Buffalo, State University of New York, Buffalo, New York, USA; **ssethi@buffalo.edu**

Fig. S1 Clinical failure rates[a] at 8 weeks post-therapy in overall and in patients with or without *P. aeruginosa* at enrolment (ITT population, N = 1352)

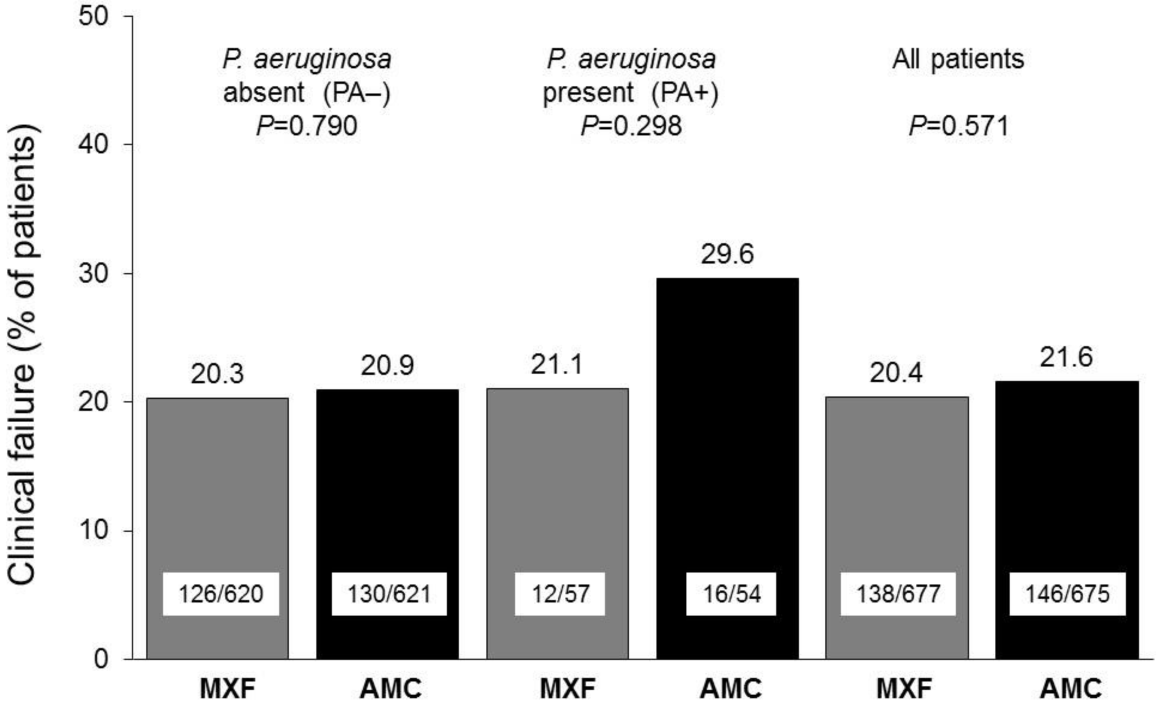

[a] Failure and relapse; AMC = amoxicillin/clavulanic acid; ITT = intent-to-treat; MXF = moxifloxacin
