# Supplementary material for: Determinants of bacteriological outcomes in exacerbations of chronic obstructive pulmonary disease
Source: Infection. 2015 Sep 14;44:65–76. doi: 10.1007/s15010-015-0833-3 (PMC4735236; doi:10.1007/s15010-015-0833-3)
Supplement: Supplementary file 2 — Supplementary material 2 (PDF 469 kb) [file 15010_2015_833_MOESM2_ESM.pdf]

Determinants of bacteriological outcomes in exacerbations of chronic obstructive pulmonary disease; *Infection*; **Sethi S** et al. Division of Pulmonary, Critical Care and Sleep Medicine, University at Buffalo, State University of New York, Buffalo, New York, USA; **ssethi@buffalo.edu**

**Supplementary Tables**

1. List of pre-therapy clinical, demographic, medical history factors with definitions included in univariate and multivariate analyses
2. Persisting bacteria up to 8 weeks post-therapy (ITT with pathogens population)
3. Superinfecting bacteria during therapy or at end of therapy (ITT population)
4. Reinfecting bacteria isolated during follow-up at 4 or 8 weeks post-therapy (ITT population)
5. Susceptibility data of superinfecting and reinfecting pathogens
6. Bacteriological success by pathogen during the study (ITT and PP with pathogens populations)

Table S1. List of pre-therapy clinical, demographic, medical history factors and results of the univariate analysis for bacteria presence prediction

| Variable/Prognostic factor | | Univariate analysis result |
|---|---|---|
| | | ***P* value** |
| **Demographic parameter** | | |
| 1 | Region[a] | >0.1 |
| 2 | Race[b] | >0.1 |
| 3 | Gender | **0.0642** |
| 4 | BMI group[c] | >0.1 |
| 5 | Age (65 yrs) | **0.0003** |
| 6 | Employment status [e] | >0.1 |
| 7 | Smoking status [e] | >0.1 |
| 8 | Alcohol [f] | **0.0461** |
| **Medical history** | | |
| 9 | Diabetes [e] | **0.095** |
| 10 | Cardiac disorders [e] | >0.1 |
| 11 | Respiratory disorders [e] | **0.0703** |
| 12 | History of cardiopulmonary disease [e] | **0.0106** |
| 13 | History of respiratory failure [e] | **0.0349** |
| 14 | Duration of chronic bronchitis [g] | >0.1 |
| **Anamnesis related to exacerbation** | | |
| 15 | Heart rate (> or ≤ 90 beats/min) | >0.1 |
| 16 | Respiration rate (> or ≤ 20/min) | >0.1 |
| 17 | Body temperature (< 36.0°C, 36-37°C, or ≥37°C) | >0.1 |
| 18 | % predicted $FEV_1$ [h] | **0.021** |
| 19 | Time since last exacerbation [i] | >0.1 |
| 20 | Number of episodes of acute exacerbation in the last 12 months [j] | >0.1 |
| 21 | Exacerbation in last 3 months [e] | **0.0688** |
| 22 | Cough frequency pre-exacerbation [k] | **0.0966** |
| 23 | Cough frequency at exacerbation [k] | >0.1 |
| 24 | Dyspnea pre-exacerbation [l] | >0.1 |
| 25 | Dyspnea at exacerbation [m] | >0.1 |

| | | |
|---|---|---|
| 26 | Wheeze pre-exacerbation [l] | **0.0468** |
| 27 | Wheeze at exacerbation [l] | **0.0327** |
| 28 | Chest discomfort pre-exacerbation [l] | **0.02** |
| 29 | Chest discomfort at exacerbation [l] | >0.1 |
| **Medications** | | |
| 30 | Co-administration of systemic corticosteroids for the current exacerbation [e] | >0.1 |
| 31 | Inhaled steroids [e] | >0.1 |
| 32 | Bronchodilator use [e] | **0.004** |
| 33 | Xanthine use [e] | **0.004** |
| 34 | LABA use [e] | >0.1 |
| 35 | SABA use [e] | **0.0804** |
| 36 | Long-acting anticholinergic use [e] | >0.1 |
| 37 | Short-acting anticholinergic use [e] | **0.0148** |
| **Sputum** | | |
| 38 | Sputum color [n] | >0.1 |
| 39 | Sputum quality [o] | >0.1 |
| 40 | Sputum volume [p] | >0.1 |
| 41 | Sputum viscosity [q] | **0.0147** |
| **AECB-SS details** | | |
| 42 | AECB-SS cough phlegm | >0.1 |
| 43 | AECB-SS color phlegm | **0.0214** |
| 44 | AECB-SS difficulty breathing | >0.1 |
| 45 | AECB-SS sleep disturbance | >0.1 |
| 46 | AECB-SS disturbed during day | **0.0888** |
| 47 | AECB-SS cough frequency | >0.1 |
| 48 | AECB-SS phlegm description | >0.1 |
| **Organisms related to acute exacerbation** | | |
| 49 | Organisms present in sputum pre-therapy [e] | NA |
| 50 | Number of organisms in sputum pre-therapy [r] | NA |
| 51 | Positive culture at EOT [e] | NA |
| 52 | Number of organisms in sputum at EOT [r] | NA |
| 53 | Resistance to study drug [e] | NA |
| **Pathogenic bacteria in sputum pre-therapy** | | |
| 54 | *H. influenzae* | NA |
| 55 | *P. aeruginosa* | NA |

| | | |
|---|---|---|
| 56 | *S. pneumoniae* | NA |
| 57 | *M. catarrhalis* | NA |
| 58 | *S. aureus* | NA |

[a] Asia Pacific, EU/Canada/South Africa, Latin America; [b] White, black, Asian, American Indian/Alaskan native, Hispanic or uncodable; [c] ≤20 mg/k/m$^2$, 21–25 mg/k/m$^2$, 26–30 mg/k/m$^2$, 31–35 mg/k/m$^2$ or >35 mg/k/m$^2$; [d] unemployed, employed, retired; [e] yes or no; [f] abstinent, light alcohol use or moderate alcohol use; [g] <10 yrs, ≤10 yrs and <20 yrs, ≤20 yrs; [h] <30 %, ≤30 % and <60 %, ≤60 %; [i] ≤2 mo, <2 mo and ≤3 mo, <3 and ≤6 mo, <6 mo; [j] 1–5; [k] none, mild, moderate or severe; [l] absent, present; [m] same as baseline, slightly increased or greatly increased; [n] clear, yellow, green, rust; [o] clear, mucoid, mucopurulent, purulent; [p] ≤5 ml, 6–15 ml, 16–30 ml, 31–50 ml, >50 ml; [q] liquid, thick, very thick, quite thick; [r] 0–4; BMI = body mass index; EOT = end of therapy; $FEV_1$ = forced expiratory volume in 1 second; AECB-SS = acute exacerbation of chronic bronchitis symptom scale; LABA = long-acting beta-2 agonist; SABA = short-acting beta-2 agonist; NA = not applicable

Table S2. Persisting bacteria up to 8 weeks post-therapy in the ITT with pathogens (N = 662) and PP with pathogens (N = 521) populations

| | **ITT with pathogens** | | **PP with pathogens** | |
|---|---|---|---|---|
| | **MXF** | **AMC** | **MXF** | **AMC** |
| | n (%) | n (%) | n (%) | n (%) |
| **Gram-positive** | | | | |
| ***Staphylococcus aureus*, n** | 23 | 20 | 18 | 17 |
| During therapy | 5 (21.7 ) | 4 (20.0) | 4 (22.2 ) | 4 (23.5) |
| EOT | 3 (13.0) | 4 (20.0) | 3 (16.7) | 4 (23.5) |
| 4 weeks | 2 (8.6) | 9 (45.0) | 2 (11.1) | 8 (47.1) |
| 8 weeks | 4 (17.4) | 6 (30.0) | 4 (22.2) | 5 (29.4) |
| ***Staphylococcus haemolyticus*, n** | 0 | 1 | 0 | 1 |
| During therapy | 0 | 1 (100) | 0 | 1 (100) |
| EOT | 0 | 0 | 0 | 0 |
| 4 weeks | 0 | 0 | 0 | 0 |
| 8 weeks | 0 | 0 | 0 | 0 |
| ***Streptococcus pneumoniae*, n** | 49 | 38 | 41 | 30 |
| During therapy | 1 (2.0) | 1 (2.6) | 1 (2.4) | 1 (3.3) |
| EOT | 5 (10.2) | 4 (10.6) | 5 (12.2) | 4 (13.3) |
| 4 weeks | 11 (22.4) | 6 (15.8) | 9 (22.0) | 5 (16.6) |
| 8 weeks | 11 (22.4) | 6 (15.8) | 9 (22.0) | 5 (16.6) |
| ***Streptococcus* spp, n** | 3 | 5 | 2 | 4 |
| During therapy | 1 (33.3) | 1 (20.0) | 0 | 1 (25.0) |
| EOT | 2 (66.7) | 1 (20.0) | 1 (50.0) | 0 |
| 4 weeks | 1 (33.3) | 1 (20.0) | 1 (50.0) | 2 (50.0) |
| 8 weeks | 1 (33.3) | 3 (60.0) | 0 | 2 (50.0) |
| ***Streptococcus pyogenes*, n** | 0 | 3 | 0 | 2 |
| During therapy | 0 | 0 | 0 | 0 |
| EOT | 0 | 0 | 0 | 0 |
| 4 weeks | 0 | 1 (33.3) | 0 | 1 (50.0) |
| 8 weeks | 0 | 2 (66.6) | 0 | 1 (50.0) |

| | | | | |
|---|---|---|---|---|
| **Gram-negative non-Enterobacteriaceae** | | | | |
| ***Pseudomonas aeruginosa*, n** | 57 | 54 | 47 | 38 |
| During therapy | 25 (43.9) | 25 (46.3) | 23 (48.9) | 19 (50.0) |
| EOT | 26 (45.6) | 19 (35.2) | 18 (38.3) | 14 (36.8) |
| 4 weeks | 21 (36.8) | 18 (33.3) | 17 (36.2) | 15 (39.8) |
| 8 weeks | 16 (28.1) | 20 (37.0) | 13 (27.7) | 18 (47.4) |
| ***Haemophilus influenzae*, n** | 65 | 75 | 53 | 56 |
| During therapy | 3 (4.6) | 9 (12.0) | 2 (3.8) | 8 (14.3) |
| EOT | 5 (7.7) | 21 (28.0) | 4 (7.6) | 14 (25.0) |
| 4 weeks | 9 (13.8) | 22 (29.4) | 8 (15.1) | 17 (30.4) |
| 8 weeks | 13 (20.0) | 27 (36.0) | 12 (22.6) | 21 (37.5) |
| ***Haemophilus parainfluenzae*, n** | 5 | 13 | 4 | 10 |
| During therapy | 1 (20.0) | 1 (7.7) | 1 (25.0) | 0 |
| EOT | 0 | 2 (15.4) | 0 | 1 (10.0) |
| 4 weeks | 0 | 2 (15.4) | 0 | 1 (10.0) |
| 8 weeks | 1 (20.0) | 3 (23.1) | 1 (25.0) | 2 (20.0) |
| ***Haemophilus* spp, n** | 2 | 1 | 2 | 1 |
| During therapy | 0 | 0 | 0 | 0 |
| EOT | 0 | 1 (100) | 0 | 1 (100) |
| 4 weeks | 1 (50.0) | 0 | 1 (50.0) | 0 |
| 8 weeks | 0 | 0 | 0 | 0 |
| ***Haemophilus haemolyticus*, n** | 1 | 0 | 1 | 0 |
| During therapy | 0 | 0 | 0 | 0 |
| EOT | 0 | 0 | 0 | 0 |
| 4 weeks | 0 | 0 | 0 | 0 |
| 8 weeks | 1 (100.0) | 0 | 1 (100.0) | 0 |
| ***Acinetobacter baumannii*, n** | 12 | 6 | 11 | 5 |
| During therapy | 2 (16.7) | 1 (16.7) | 2 (18.2) | 1 (20.0) |
| EOT | 2 (16.7) | 2 (33.3) | 1 (9.1) | 2 (40.0) |
| 4 weeks | 4 (33.3) | 3 (50.0) | 0 | 1 (20.0) |
| 8 weeks | 3 (25.0) | 0 | 3 (27.3) | 0 |
| ***Acinetobacter lwoffii*, n** | 3 | 1 | 3 | 1 |

| | | | | |
|---|---|---|---|---|
| During therapy | 1 (33.3) | 0 | 1 (33.3) | 0 |
| EOT | 0 | 1 (100.0) | 0 | 1 (100.0) |
| 4 weeks | 0 | 0 | 0 | 0 |
| 8 weeks | 1 (33.3) | 0 | 1 (33.3) | 0 |
| ***Moraxella catarrhalis*, n** | 36 | 43 | 26 | 35 |
| During therapy | 1 (2.8) | 5 (11.6) | 1 (3.8) | 5 (14.3) |
| EOT | 4 (11.1) | 3 (7.0) | 4 (15.4) | 2 (5.7) |
| 4 weeks | 7 (19.4) | 6 (13.9) | 4 (15.4) | 6 (17.1) |
| 8 weeks | 12 (33.3) | 8 (18.6) | 9 (34.6) | 7 (20.0) |
| ***Stenotrophomonas maltophilia*, n** | 2 | 6 | 1 | 6 |
| During therapy | 1 (50.0) | 1 (16.7) | 1 (100.0) | 1 (16.7) |
| EOT | 0 | 2 (33.3) | 0 | 2 (33.3) |
| 4 weeks | 0 | 2 (33.3) | 0 | 2 (33.3) |
| 8 weeks | 0 | 2 (33.3) | 0 | 2 (33.3) |
| ***Morganella morganii*, n** | 3 | 0 | 2 | 0 |
| During therapy | 0 | 0 | 0 | 0 |
| EOT | 1 (33.3) | 0 | 0 | 0 |
| 4 weeks | 1 (33.3) | 0 | 0 | 0 |
| 8 weeks | 1 (33.3) | 0 | 0 | 0 |
| ***Burkholderia cepacia*, n** | 1 | 1 | 1 | 1 |
| During therapy | 1 (100.0) | 0 | 1 (100.0) | 0 |
| EOT | 1 (100.0) | 1 (100.0) | 1 (100.0) | 1 (100.0) |
| 4 weeks | 1 (100.0) | 1 (100.0) | 1 (100.0) | 1 (100.0) |
| 8 weeks | 1 (100.0) | 1 (100.0) | 1 (100.0) | 1 (100.0) |
| ***Alcaligenes xylosoxidans*, n** | 0 | 1 | 0 | 1 |
| During therapy | 0 | 0 | 0 | 0 |
| EOT | 0 | 1 (100.0) | 0 | 1 (100.0) |
| 4 weeks | 0 | 0 | 0 | 0 |
| 8 weeks | 0 | 0 | 0 | 0 |
| **Enterobacteriaceae** | | | | |
| ***Klebsiella pneumoniae*, n** | 36 | 48 | 26 | 43 |
| During therapy | 6 (16.7) | 24 (50.0) | 5 (19.2) | 22 (51.2) |

| | | | | |
|---|---|---|---|---|
| EOT | 12 (33.3) | 17 (35.4) | 9 (34.6) | 14 (32.6) |
| 4 weeks | 12 (33.3) | 11 (22.9) | 8 (30.8) | 10 (23.3) |
| 8 weeks | 9 (25.0) | 12 (25.0) | 8 (30.8) | 11 (25.6) |
| ***Klebsiella oxytoca*, n** | 11 | 4 | 11 | 1 |
| During therapy | 1 (9.1) | 2 (50.0) | 1 (9.1) | 1 (100.0) |
| EOT | 2 (18.2) | 1 (25.0) | 2 (18.2) | 0 |
| 4 weeks | 0 | 0 | 0 | 0 |
| 8 weeks | 0 | 0 | 0 | 0 |
| ***Escherichia coli*, n** | 21 | 16 | 16 | 12 |
| During therapy | 3 (14.3) | 6 (37.5) | 2 (12.5) | 5 (41.7) |
| EOT | 6 (28.6) | 4 (25.0) | 5 (31.3) | 4 (33.3) |
| 4 weeks | 6 (28.6) | 6 (37.5) | 5 (31.3) | 4 (33.3) |
| 8 weeks | 9 (42.9) | 7 (43.8) | 7 (43.8) | 5 (41.7) |
| ***Enterobacter cloacae*, n** | 11 | 8 | 10 | 5 |
| During therapy | 0 | 3 (37.5) | 0 | 1 (20.0) |
| EOT | 2 (18.2) | 0 | 2 (20.0) | 0 |
| 4 weeks | 2 (18.2) | 0 | 2 (20.0) | 0 |
| 8 weeks | 3 (27.3) | 0 | 3 (30.0) | 0 |
| ***Enterobacter aerogenes*, n** | 8 | 8 | 6 | 6 |
| During therapy | 1 (12.5) | 3 (37.5) | 1 (16.7) | 3 (50.0) |
| EOT | 2 (25.0) | 2 (25.0) | 2 (33.3) | 2 (33.3) |
| 4 weeks | 1 (12.5) | 3 (37.5) | 1 (16.7) | 1 (16.7) |
| 8 weeks | 1 (12.5) | 5 (62.5) | 1 (16.7) | 2 (33.3) |
| ***Serratia marcescens*, n** | 14 | 14 | 11 | 12 |
| During therapy | 3 (21.4) | 7 (50.0) | 2 (18.2) | 6 (50.0) |
| EOT | 4 (28.6) | 4 (28.6) | 3 (27.3) | 3 (25.0) |
| 4 weeks | 2 (14.2) | 3 (21.4) | 1 (9.1) | 3 (25.0) |
| 8 weeks | 3 (21.4) | 3 (21.4) | 2 (18.2) | 3 (25.0) |
| ***Proteus mirabilis*, n** | 4 | 9 | 2 | 8 |
| During therapy | 2 (50.0) | 3 (33.3) | 2 (100.0) | 8 (100.0) |
| EOT | 3 (75.0) | 3 (33.3) | 2 (100.0) | 3 (37.5) |
| 4 weeks | 2 (50.0) | 4 (44.4) | 2 (100.0) | 4 (50.0) |

| | | | | |
|---|---|---|---|---|
| 8 weeks | 2 (50.0) | 5 (55.5) | 2 (100.0) | 5 (62.5) |
| ***Proteus vulgaris*, n** | 0 | 2 | 0 | 2 |
| During therapy | 0 | 1 (50.0) | 0 | 1 (50.0) |
| EOT | 0 | 0 | 0 | 0 |
| 4 weeks | 0 | 0 | 0 | 0 |
| 8 weeks | 0 | 0 | 0 | 0 |
| ***Citrobacter freundii*, n** | 1 | 1 | 1 | 1 |
| During therapy | 1 (100.0) | 0 | 1 (100.0) | 0 |
| EOT | 0 | 1 (100.0) | 0 | 1 (100.0) |
| 4 weeks | 0 | 1 (100.0) | 0 | 1 (100.0) |
| 8 weeks | 0 | 1 (100.0) | 0 | 1 (100.0) |
| ***Citrobacter koseri*, n** | 6 | 7 | 5 | 5 |
| During therapy | 2 (33.3) | 5 (71.4) | 2 (40.0) | 4 (80.0) |
| EOT | 3 (50.0) | 6 (85.7) | 3 (60.0) | 4 (50.0) |
| 4 weeks | 2 (33.3) | 5 (71.4) | 1 (20.0) | 3 (60.0) |
| 8 weeks | 2 (33.3) | 5 (71.4) | 1 (20.0) | 3 (60.0) |
| ***Citrobacter amalonaticus*, n** | 1 | 1 | 1 | 1 |
| During therapy | 0 | 1 (100.0) | 0 | 1 (100.0) |
| EOT | 0 | 0 | 0 | 0 |
| 4 weeks | 0 | 0 | 0 | 0 |
| 8 weeks | 0 | 1 (100.0) | 0 | 1 (100.0) |

number of patients with persisting pathogens; AMC = amoxicillin/clavulanic acid; EOT = end of therapy; ITT = intent-to-treat; MXF = moxifloxacin; PP = per-protocol

Table S3. Superinfecting bacteria during therapy or at end of therapy (ITT population)

| | **Total N=1352** | **MXF N=677** | **AMC N=675** |
|---|---|---|---|
| | **n (%)** | **n (%)** | **n (%)** |
| **Any** | 148 (10.9) | 65 (9.6) | 83 (12.3) |
| *Staphylococcus aureus* | 17 (1.3) | 9 (1.3) | 8 (1.2) |
| *Streptococcus pneumoniae* | 14 (1.0) | 5 (0.7) | 9 (1.3) |
| *Streptococcus* spp. | 4 (0.3) | 4 (0.6) | 0 (-) |
| *Enterococcus faecalis* | 4 (0.3) | 3 (0.4) | 1 (0.15) |
| *Streptococcus agalactiae* | 2 (0.15) | 2 (0.3) | 0 (-) |
| *Streptococcus anginosus* | 2 (0.15) | 1 (0.15) | 1 (0.15) |
| *Staphylococcus haemolyticus* | 2 (0.15) | 2 (0.3) | 0 (-) |
| *Streptococcus sanguis* | 1 (0.07) | 1 (0.15) | 0 (-) |
| *Streptococcus viridans* | 1 (0.07) | 0 (-) | 1 (0.15) |
| *Pseudomonas aeruginosa* | 45 (3.3) | 20 (3.0) | 25 (3.7) |
| *Haemophilus influenzae* | 24 (1.8) | 6 (0.9) | 18 (2.7) |
| *Acinetobacter baumannii* | 22 (1.6) | 13 (1.9) | 9 (1.3) |
| *Moraxella catarrhalis* | 20 (1.5) | 10 (1.5) | 10 (1.5) |
| *Haemophilus parainfluenzae* | 9 (0.7) | 4 (0.6) | 5 (0.7) |
| *Stenotrophomonas maltophilia* | 5 (0.4) | 2 (0.3) | 3 (0.4) |
| *Acinetobacter lwoffii* | 2 (0.15) | 1 (0.15) | 1 (0.15) |
| *Morganella morganii* | 2 (0.15) | 0 (-) | 2 (0.3) |
| *Haemophilus parahaemolyticus* | 2 (0.15) | 1 (0.15) | 1 (0.15) |
| *Haemophilus haemolyticus* | 1 (0.07) | 0 (-) | 1 (0.15) |
| *Burkholderia cepacia* | 1 (0.07) | 0 (-) | 1 (0.15) |
| *Klebsiella pneumoniae* | 36 (2.7) | 14 (2.1) | 22 (3.3) |
| *Enterobacter cloacae* | 18 (1.3) | 4 (0.6) | 14 (2.1) |
| *Escherichia coli* | 18 (1.3) | 8 (1.2) | 10 (1.5) |
| *Klebsiella oxytoca* | 15 (1.1) | 1 (0.15) | 14 (2.1) |
| *Serratia marcescens* | 13 (1.0) | 5 (0.7) | 8 (1.2) |
| *Proteus mirabilis* | 7 (0.5) | 4 (0.6) | 3 (0.4) |
| *Enterobacter aerogenes* | 7 (0.5) | 1 (0.15) | 6 (0.9) |

| | | | |
|---|---|---|---|
| *Citrobacter freundii* | 4 (0.3) | 1 (0.15) | 3 (0.4) |
| *Citrobacter koseri* | 3 (0.2) | 1 (0.15) | 2 (0.3) |
| *Proteus vulgaris* | 3 (0.2) | 2 (0.3) | 1 (0.15) |
| *Citrobacter amalonaticus* | 1 (0.07) | 0 (-) | 1 (0.15) |
| *Klebsiella ozaenae* | 1 (0.07) | 0 (-) | 1 (0.15) |
| *Serratia odorifera* | 1 (0.07) | 0 (-) | 1 (0.15) |

number of patients with superinfection; AMC = amoxicillin/clavulanic acid; ITT = intent-to-treat; MXF = moxifloxacin

Table S4. Reinfecting bacteria isolated at 4 or 8 weeks post-therapy (ITT population)

| | **Total N = 1352** | **MXF N = 677** | **AMC N = 675** |
|---|---|---|---|
| | **n (%)** | **n (%)** | **n (%)** |
| **Any** | 119 (8.8) | 58 (8.6) | 61 (9.0) |
| *Streptococcus pneumoniae* | 27 (2.0) | 11 (1.6) | 16 (2.4) |
| *Staphylococcus aureus* | 9 (0.7) | 7 (1.0) | 2 (0.3) |
| *Enterococcus faecalis* | 2 (0.15) | 2 (0.3) | 0 (-) |
| *Streptococcus agalactiae* | 2 (0.15) | 2 (0.3) | 0 (-) |
| *Streptococcus anginosus* | 1 (0.07) | 0 (-) | 1 (0.15) |
| *Streptococcus* spp. | 1 (0.07) | 1 (0.15) | 0 (-) |
| *Pseudomonas aeruginosa* | 39 (2.9) | 21 (3.1) | 18 (2.7) |
| *Haemophilus influenzae* | 26 (1.9) | 7 (1.0) | 19 (2.8) |
| *Moraxella catarrhalis* | 24 (1.8) | 16 (2.4) | 8 (1.2) |
| *Acinetobacter baumannii* | 8 (0.6) | 4 (0.6) | 4 (0.6) |
| *Haemophilus parainfluenzae* | 4 (0.3) | 3 (0.4) | 1 (0.15) |
| *Morganella morganii* | 4 (0.3) | 3 (0.4) | 1 (0.15) |
| *Stenotrophomonas maltophilia* | 3 (0.2) | 2 (0.3) | 1 (0.15) |
| *Acinetobacter lwoffii* | 1 (0.07) | 0 (-) | 1 (0.15) |
| *Burkholderia cepacia* | 1 (0.07) | 1 (0.15) | 0 (-) |
| *Haemophilus parahaemolyticus* | 1 (0.07) | 0 (-) | 1 (0.15) |
| *Haemophilus* spp. | 1 (0.07) | 0 (-) | 1 (0.15) |
| *Moraxella* spp. | 1 (0.07) | 0 (-) | 1 (0.15) |
| *Klebsiella pneumoniae* | 29 (2.1) | 14 (2.1) | 15 (2.2) |
| *Klebsiella oxytoca* | 16 (1.2) | 5 (0.7) | 11 (1.6) |
| *Escherichia coli* | 12 (0.9) | 8 (1.2) | 4 (0.6) |
| *Enterobacter cloacae* | 12 (0.9) | 3 (0.4) | 9 (1.3) |
| *Serratia marcescens* | 11 (0.8) | 6 (0.9) | 5 (0.7) |
| *Enterobacter aerogenes* | 8 (0.6) | 5 (0.7) | 3 (0.4) |
| *Citrobacter koseri* | 5 (0.4) | 2 (0.3) | 3 (0.4) |
| *Proteus mirabilis* | 3 (0.2) | 1 (0.15) | 2 (0.3) |
| *Citrobacter freundii* | 2 (0.15) | 1 (0.15) | 1 (0.15) |

| | | | |
|---|---|---|---|
| *Klebsiella ozaenae* | 1 (0.07) | 1 (0.15) | 0 (-) |

number of patients with reinfection; AMC = amoxicillin/clavulanic acid; ITT = intent-to-treat; MXF = moxifloxacin

Table S5. Susceptibility data of superinfecting bacteria (ITT population)

| **Organism** | **Total number** | **MXF** | | **AMC** | |
|---|---|---|---|---|---|
| | | **N** | **Range (mg/L)** | **N** | **Range (mg/L)** |
| *Klebsiella pneumoniae*[a] | 24 | 10 | 0.06 –8.0 | 14 | 1.0–64.0 |
| *Pseudomonas aeruginosa*[b] | 21 | 12 | 0.5–4.0 | 9 | 32.0–64.0 |
| *Escherichia coli* | 11 | 5 | 0.03–8.0 | 6 | 4.0–8.0 |
| *Moraxella catarrhalis* | 10 | 6 | 0.03–0.06 | 4 | 0.06–0.5 |
| *Klebsiella oxytoca*[c] | 9 | - | - | 9 | 1.0–64.0 |
| *Staphylococcus aureus* | 8 | 5 | 0.03–2.0 | 3 | 0.5–1.0 |
| *Haemophilus influenzae* | 8 | 1 | 0.015 | 7 | 1.0–2.0 |
| *Enterobacter cloacae* | 8 | 2 | 0.12–0.25 | 6 | 64.0–64.0 |
| *Serratia marcescens* | 7 | 3 | 0.06–8.0 | 4 | 8.0–64.0 |
| *Streptococcus pneumoniae* | 6 | 3 | 0.012–2.0 | 3 | 2.0–4.0 |
| *Proteus mirabilis* | 5 | 3 | 0.5–8.0 | 2 | 1.0–64.0 |
| *Citrobacter freundii* | 3 | - | - | 3 | 16.0-64.0 |
| *Enterobacter aerogenes* | 2 | 1 | 0.12 | 1 | 64 |
| *Citrobacter koseri* | 2 | 1 | 0.06 | 1 | 2 |
| *Acinetobacter baumannii* | 1 | 1 | 0.12 | - | - |
| *Proteus vulgaris* | 1 | - | - | 1 | 4 |
| *Serratia odorifera* | 1 | - | - | 1 | 2 |
| *Morganella morganii* | 1 | - | - | 1 | 2 |
| *Burkholderia cepacia* | 1 | - | - | 1 | 2 |
| *Haemophilus parainfluenzae* | 1 | - | - | 1 | 1 |

[a] MXF $MIC_{50}$ = 0.12 mg/L, $MIC_{90}$ = 0.25 mg/L; AMC $MIC_{50}$ = 2 mg/L, $MIC_{90}$ = 4 mg/L; [b] MXF $MIC_{50}$ = 2 mg/L, $MIC_{90}$ = 4 mg/L; AMC $MIC_{50}$ = 64 mg/L; [c] AMC $MIC_{50}$ = 2 mg/L; AMC = amoxicillin/clavulanic acid; ITT = intent-to-treat; MXF = moxifloxacin

Table S6. Bacteriological success[a] by pathogen at the different time points (ITT and PP with pathogens populations)

| | **ITT with pathogens** | | **PP with pathogens** | |
|---|---|---|---|---|
| | **MXF** | **AMC** | **MXF** | **AMC** |
| | n (%) | n (%) | n (%) | n (%) |
| ***Pseudomonas aeruginosa*, n** | 57 | 54 | 47 | 38 |
| During therapy | 31 (54.4) | 28 (51.9) | 24 (51.1) | 18 (47.4) |
| EOT | 31 (54.4) | 32 (59.3) | 29 (61.7) | 22 (57.9) |
| 4 weeks | 31 (54.4) | 33 (61.1) | 29 (61.7) | 23 (60.5) |
| 8 weeks | 36 (63.2) | 28 (51.9) | 34 (72.3) | 20 (52.6) |
| | ***P* = 0.228** | | ***P* = 0.061** | |
| ***Streptococcus pneumoniae*, n** | 49 | 38 | 41 | 30 |
| During therapy | 48 (98) | 35 (92.1) | 40 (97.6) | 29 (96.7) |
| EOT | 44 (89.8) | 33 (86.8) | 36 (87.8) | 26 (86.7) |
| 4 weeks | 36 (73.5) | 29 (76.3) | 32 (78.0) | 25 (83.3) |
| 8 weeks | 37 (75.5) | 27 (71.1) | 32 (78.0) | 25 (83.3) |
| | ***P* = 0.640** | | ***P* = 0.580** | |
| ***Klebsiella pneumoniae*, n** | 36 | 48 | 26 | 43 |
| During therapy | 28 (77.8) | 22 (45.8) | 21 (80.8) | 20 (46.5) |
| EOT | 21 (58.3) | 29 (60.4) | 17 (65.4) | 28 (65.1) |
| 4 weeks | 19 (52.8) | 34 (70.8) | 16 (61.5) | 33 (76.7) |
| 8 weeks | 22 (61.1) | 33 (68.8) | 18 (69.2) | 32 (74.4) |
| | ***P* = 0.466** | | ***P* = 0.640** | |
| ***Moraxella catarrhalis*, n** | 36 | 43 | 26 | 35 |
| During therapy | 32 (88.9) | 36 (83.7) | 25 (96.2) | 30 (85.7) |
| EOT | 30 (83.3) | 37 (86.0) | 22 (84.6) | 33 (94.3) |
| 4 weeks | 25 (69.4) | 34 (79.1) | 21 (80.8) | 29 (82.9) |
| 8 weeks | 21 (58.3) | 33 (76.7) | 17 (65.4) | 28 (80.0) |
| | ***P* = 0.080** | | ***P* = 0.199** | |
| ***Staphylococcus aureus*, n** | 23 | 20 | 18 | 17 |
| During therapy | 18 (78.3 ) | 16 (80.0) | 14 (77.8) | 13 (76.5) |

| | | | | |
|---|---|---|---|---|
| EOT | 20 (87.0) | 16 (80.0) | 15 (83.3) | 13 (76.5) |
| 4 weeks | 21 (91.3) | 11 (55.0) | 16 (88.9) | 9 (52.9) |
| 8 weeks | 18 (78.3) | 14 (70.0) | 14 (77.8) | 12 (70.6) |
| | ***P* = 0.536** | | ***P* = 0.627** | |
| ***Escherichia coli*, n** | 21 | 16 | 16 | 12 |
| During therapy | 17 (81.0) | 10 (62.5) | 14 (87.5) | 7 (58.3) |
| EOT | 14 (66.7) | 11 (68.8) | 11 (68.8) | 8 (66.7) |
| 4 weeks | 14 (66.7) | 10 (62.5) | 11 (68.8) | 8 (66.7) |
| 8 weeks | 11 (52.4) | 9 (56.3) | 9 (56.3) | 7 (58.3) |
| | ***P* = 0.815** | | ***P* = 0.912** | |

[a] Eradication + presumed eradication; AMC = amoxicillin/clavulanic acid; EOT = end of therapy;

ITT = intent-to-treat; MXF = moxifloxacin; PP = per-protocol
